# Lateral Pelvic Wall Schwannoma: A Case Report and Literature Review

**DOI:** 10.7759/cureus.99569

**Published:** 2025-12-18

**Authors:** David Chou, Assad Zahid, Ernest Cheng

**Affiliations:** 1 General Surgery, Liverpool Hospital, Liverpool, AUS; 2 General Surgery, Campbelltown Hospital, Sydney, AUS

**Keywords:** abdominal schwannoma, histopathological diagnosis, neurilemma, pelvic cavity, pelvic wall schwannoma, various surgical procedures

## Abstract

Schwannomas are a type of benign nerve sheath tumor originating from Schwann cells that very rarely originate from nerves within the pelvis. Here, we report a case of a 65-year-old male with a background of rectal adenocarcinoma who had a lesion identified within his right lateral pelvic wall on an MRI scan, initially believed to be metastatic spread to a lymph node. He underwent a laparoscopic abdominoperineal resection as well as a lateral pelvic side wall dissection. Histopathology identified the lesion as a schwannoma, which, upon review of the recorded laparoscopic footage, was identified as originating from the obturator nerve.

## Introduction

Schwannomas are the most common subtype of peripheral nerve tumors. They are formed from benign neoplastic Schwann cells, developing from nerve roots or peripheral nerves in a sporadic fashion [1]. Schwannomas mainly show two histological types, Antoni A and B, where Antoni A are described as having “dense” compact, elongated cells, while Antoni B are “loose,” disorganized with sparse cells [1]. They are slow growing, with most patients presenting as asymptomatic and only a small proportion showing symptoms of pain and sensory disturbances [1]. While most schwannomas involve nerves within the head, neck, limbs, and extremities, there have been rare cases of them developing as isolated tumors within the pelvis [2-6], with a variety of symptoms, including lower back discomfort [2], abdominal distension [4], pain [5], sphincter problems, and leg weakness [6]. We present a rare case of a right lateral pelvic schwannoma identified on an MRI scan, where the final diagnosis was confirmed with laparoscopic surgery and histopathology.

## Case presentation

**Figure 1:**
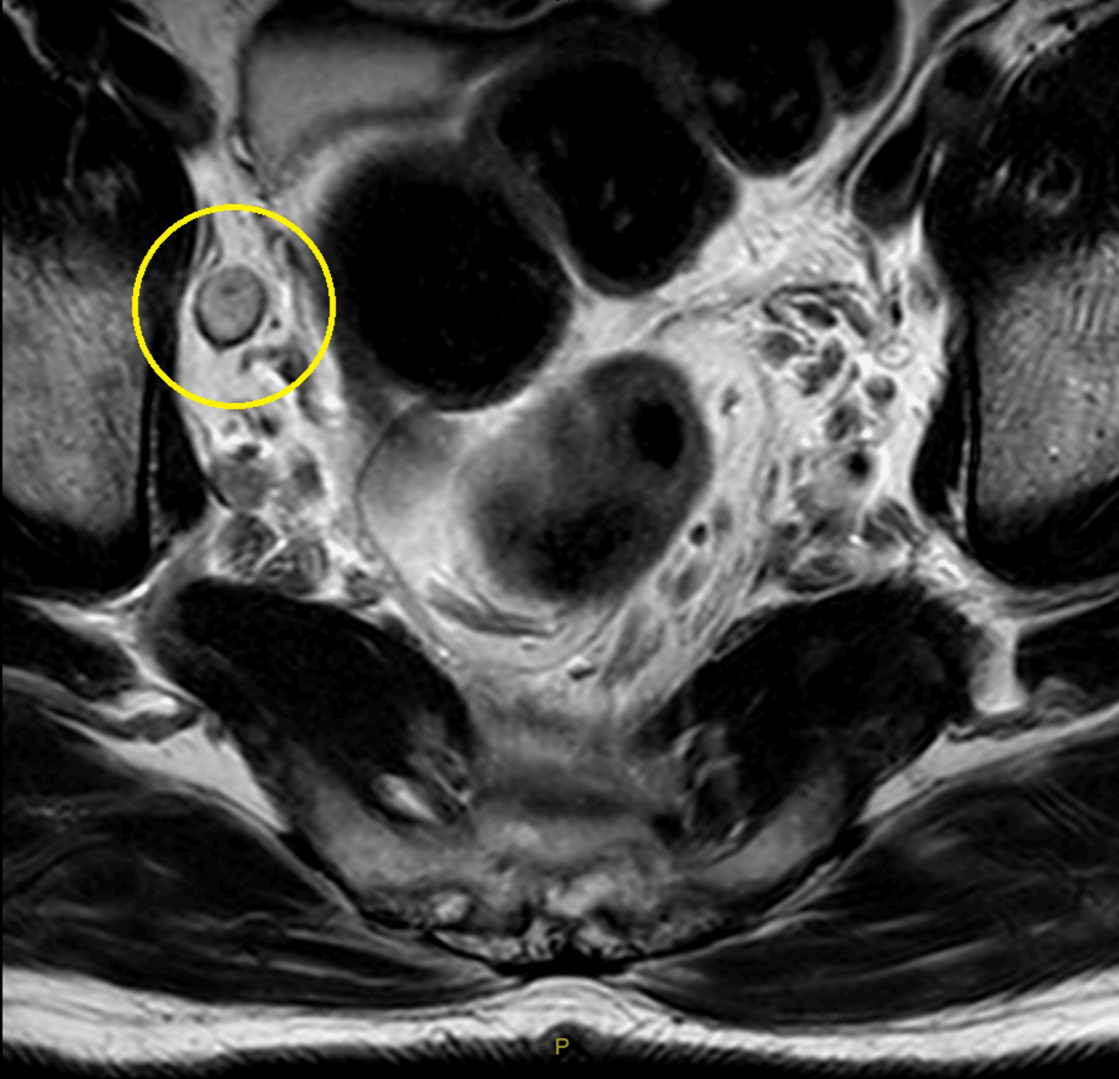
MRI scan of the pelvis with marked region highlighting a right pelvic side wall node.

A 65-year-old male was admitted to our hospital for an elective procedure to excise a known low rectal adenocarcinoma, for which he had been undergoing chemotherapy for the past 11 months with good response. On his latest progress MRI scan, it was noted that his primary tumor had decreased in size and was suitable for resection. A right pelvic side wall node was also identified (Figure 1). The decision was made to perform a laparoscopic abdominal-perineal resection for his primary tumor as well as a dissection of the right side of his pelvis to remove the suspected node.

The patient was placed into lithotomy position under general anesthesia, and an infraumbilical 10 mm port was inserted with the Hassan technique. Four additional 5 mm ports were inserted in the right upper quadrant, left upper quadrant, suprapubic, and left lower quadrant areas. After the abdominal-perineal resection was performed with an intersphincteric dissection, a right lateral pelvic dissection was performed with laparoscopic scissors and a Harmonic© scalpel (Ethicon, Raritan, NJ) (Video 1). A grey/tan nodule was identified in the lateral pelvic space, which was dissected off the pelvic side wall. As the nature of the nodule had yet to be identified at the time, the decision was made to isolate the nodule from the attached structures with a Ligaclip© multi-clip applier (Ethicon) with two clips on either side of the nodule. The nodule was subsequently excised with the Harmonic© scalpel on the medial side of the clips, closer to the nodule.

**Video 1:** Dissection of the right lateral pelvic wall node, performed laparoscopically.

**Figure 2:**
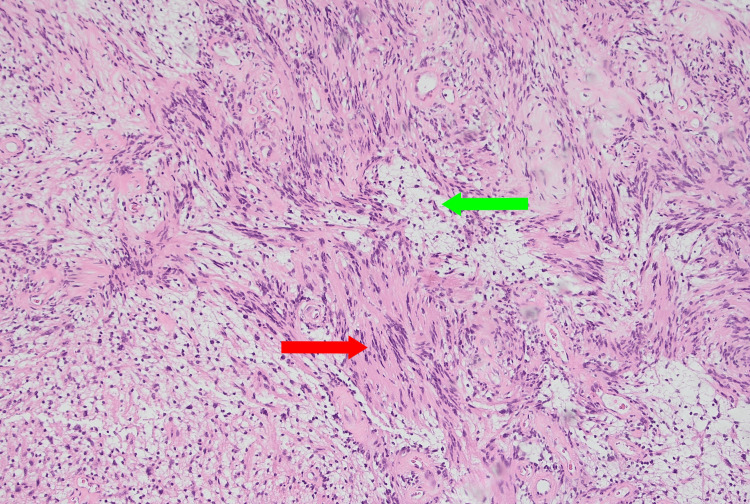
High-power view of a section of the microscopic slide of the schwannoma. This specimen shows all typical microscopic features of a schwannoma, including dense, compact, spindle-shaped Antoni A cells (red arrow) with their characteristic Verocay bodies [1], and loose, disorganized, and sparse Antoni B cells (green arrow) [1].

On macroscopic pathological assessment, it was noted to be a 13 x 12 x 6 mm firm fibrous nodule with a capsule, believed to be a malignant lymph node. Further histopathology identified the nodule as a schwannoma (Figure 2). On further inspection of the recorded video of the operation, the schwannoma was identified to have formed from the obturator nerve. However, on later follow-up with the patient, he denied any neurological deficits. This has been attributed to the patient's poor baseline mobility secondary to his significant comorbidities, which has potentially resulted in his deficits not being as pronounced.

## Discussion

While schwannomas are already rare, retroperitoneal schwannomas (which include pelvic schwannomas) are noted to be even rarer, with estimates of 3% of all schwannomas [3]. These kinds of schwannomas tend to arise from the peripheral nerve sheath of the hypogastric plexus or sacral nerve, and nearly all are benign [4]. Most patients with peripheral nerve lesions present as asymptomatic; however, there have been cases of schwannomas that involve spinal nerve roots presenting with nerve compression symptoms and radicular pain.

Diagnosis of schwannomas perioperatively tends to be performed via radiological assessment, such as ultrasound, CT, or MRI; however, this is noted to be very difficult [7]. MRI scans are notably an important method for diagnosis, as they are capable of determining the tumor’s point of origin, defining margins, as well as identifying any cystic degeneration [8]. Schwannomas are typically defined by two types of characteristic signals on MRI: a T1 hyposignal identical to the consistency of adjacent skeletal muscle, as well as a T2 hypersignal similar to fat [9]. However, not all schwannomas present with these findings, with their presence only present in 57% of cases. Other potential perioperative diagnostic methods include fine needle biopsy [10]; however, this is generally not recommended due to the risks of dissemination of potentially malignant tumors [2]. In our case report, due to the existing presence of recurrent metastatic rectal adenocarcinoma, as well as the small size of the schwannoma on imaging, the schwannoma was mistakenly identified as a potential spread of metastatic disease to local retroperitoneal lymph nodes, highlighting the difficulty of perioperative diagnosis in varying settings.

Surgical resection is the gold standard of treatment for schwannomas, with very few cases of reported recurrence after resection. Despite their rarity, there have been increasing reports of pelvic schwannomas appearing in the literature over the past decade, all of varying sizes. These schwannomas have been reportedly resected by a variety of specialties, including orthopedics, urology, colorectal, as well as general surgery [10]. Depending on the size of the schwannoma, the approach tends to vary, with larger lesions being investigated and removed via explorative laparotomy, while smaller lesions are removed via a laparoscopic approach. Another case in the literature has also reported the technique of robotic-assisted surgery in two patients, which resulted in excellent outcomes and a complete lack of neurological deficit [5].

If the schwannoma is identified prior to the operation, surgery should aim to preserve the nerve. Unfortunately, in our case, as the schwannoma was considered a metastatic lymph node intraoperatively, the decision was made to clamp off and completely excise the lesion, bisecting the nerve in the process. There have been cases in the literature where neurological disorders have occurred based on the location of the affected nerve [11], with obturator nerve injuries causing symptoms such as groin pain, weakness in leg adduction, as well as gait changes [12]. However, in our case, our patient did not report any noticeable neurological deficits upon later review three months post surgery. This has been attributed to our patient's poor baseline mobility secondary to his significant comorbidities, potentially leading to a deficit that does not present as pronounced.

## Conclusions

Schwannomas within the retroperitoneal space are extremely rare, and making a diagnosis perioperatively via imaging alone can be very challenging. The gold standard of treatment of schwannomas remains surgical excision, which can be performed by a variety of surgical specialties with varying techniques. The presence of pre-established metastatic disease can also make diagnosis intraoperatively difficult, which further results in differences in approach during the surgical procedure.
